# The Influence of a Novel Hydrophobic Agent on the Internal Defect and Multi-Scale Pore Structure of Concrete

**DOI:** 10.3390/ma14030609

**Published:** 2021-01-28

**Authors:** Bo Zhang, Qingbin Li, Rui Ma, Xujing Niu, Lin Yang, Yu Hu, Jinliang Zhang

**Affiliations:** 1 State Key Laboratory of Hydroscience and Engineering, Tsinghua University, Beijing 100084, China; zbdth@mail.tsinghua.edu.cn (B.Z.); qingbinli@tsinghua.edu.cn (Q.L.); marui14@tsinghua.org.cn (R.M.); wonderfulnxj@tsinghua.edu.cn (X.N.); 2 Yellow River Engineering Consulting Co., Ltd., Zhengzhou 450003, China; zzuyanglin@163.com; 3 School of Water Conservancy Engineering, Zhengzhou University, Zhengzhou 450052, China

**Keywords:** hydrophobic agent, internal defect, impermeability, strength, pore size distribution

## Abstract

In high humidity areas, it is necessary to improve the impermeability of concrete to water and other erosion solutions. The internal defect and pore channel of concrete are the main factors affecting the impermeability and durability. In this paper, a novel hydrophobic agent named Yellow River Engineering Consulting (YREC) was prepared. The relative internal defect degree of concrete with different curing ages and YREC contents was evaluated by ultrasonic non-destructive testing as qualitative characterization method, and the effect of YREC on hydration reaction was investigates using X-ray powder diffraction (XRD). Water permeability and contact angle tests were used to analyze the internal and external hydrophobicity induced by YREC addition, respectively. The pore structure changes of concrete mortar matrix induced by YREC were further discussed applying low-temperature liquid nitrogen adsorption (LT-NA) and mercury intrusion/extrusion porosimetry (MIP). The results indicated that YREC not only improves the impermeability of water, but also greatly enhances the mechanical strength. In the case of mixing YREC, the porosity of concrete mortar matrix decreases accompanied with the more advantage pores (micropores and transition pores) developed. Based on the relative internal defect degree and the changes of multi-scale pore structure, the functionality and durability of concrete with 4% YREC addition are the most desirable.

## 1. Introduction

Concrete is the most basic and important engineering material in modern society [1,2]. The utilization of low-heat Portland cement is widely considered to an effective method to decrease the hydration heat, shrinkage behavior, and cracking risk in the dam concrete [3,4,5]. Low-heat Portland cement is a greener material with lower energy consumption and lower CO_2_ emission during the production, which has been attracted much attention in the field of building materials [6,7]. The porous concrete materials generally contain various pore size distribution (PSD) characteristics and cracks, these structural defects facilitate the intrusion of water and other harmful solutions [8,9,10,11]. In terms of higher humidity area, increasing the impermeability of concrete is a necessary method to ensure its durability and functionality.

At present, some research foundation has been established to improve concrete durability by isolating concrete from external water through hydrophobic technology. The common method is surface coating that provides the concrete surface a protective layer, which can resist the intrusion of water [12]. Hydrophobic impregnation is another surface treatment, which is widely used in engineering due to the least influence on mechanical properties of concrete, and this method will not delay the progress of the project [13]. It is generally considered that hydrophobic impregnation method can provide an effective protection layer for concrete, which improve the impermeability of concrete and further protect the internal steel structure from water-dissolved salts [14,15]. Silane and siloxane materials are the primary choice for protecting concrete, and most research has focused on testing these materials and improving their performance [16,17,18,19]. Moreover, once the waterproof coating is damaged, water will still invade the concrete. In order to better solve the problem of water intrusion, it is available to make the inside and outside of concrete hydrophobic.

A better approach is to make concrete materials both internally and externally hydrophobic, and the hydrophobic admixture has been mixed in the concrete directly. Hartmut Herb et al. [20] optimized the structure of alkyltrialkoxysilanes in terms of water-repellent performance and investigated characterization of alkyltrialkoxysilanes and the corresponding reaction products in concrete. Singh et al. [21] proposed that the addition of ZnO in cement can reduce the water absorption. Tittarelli and Moriconi [22] studied the effect of silane admixture mixed in concrete and proved that silane could refrain corrosion process to some extent in uncracked concrete specimens. Di Mundo [23] showed that the addition of tyre rubber in cement matrix obviously blocked penetration of water, and the hydrophobicity performances increased with the smaller tyre rubber grains. Incorporation of polymeric fibers as filler in the concrete mixture, in combination with a waterproof coating method, has been demonstrated to abate water penetration effectively, and this technique can produce a new and progressive hydrophobicity material [24,25].

In the case of similar coarse aggregate properties, the mechanical properties of concrete materials are directly bound up with the microscopic characteristics (different pore types, various pore sizes, and micro-defects) of the mortar matrix [9,26,27,28,29]. It is generally accepted that the most variable section of concrete materials is the mortar matrix, which is also named the concrete matrix surrounded by the coarse aggregates [30]. Hydrophobic admixtures usually have hydrophobic functional groups in chemical composition or in the products of chemical reactions. One of the main factors affecting the mechanism by which water passes through the concrete is the pore structure. In physics, there are relatively few studies on the change of PSD of mortar matrix by hydrophobic admixtures. For cement based materials, many researchers also focused on the crystalline technology and self-healing capacity in order to fill or reduce pores and mic-crack, which and achieved in reducing water absorption and chloride diffusion in concrete [31,32,33]. The porosity, PSD, type, spatial distribution, and compressibility of pores have a significant effect on the durability and mechanical characteristics of concrete or mortar matrix [34]. For the characterization of cement-based materials properties, it is necessary to obtain the information of pore structure accurately. The PSD of cement-based materials was investigated by using cycling mercury intrusion/extrusion porosimetry (MIP) [34]. Pipilikaki et al. [35] studied the pore structure of cement samples by means of MIP combined with non-destructive testing method (nuclear magnetic resonance), and the characteristics of the two methods were evaluated comprehensively. The larger scale of pore sizes of high performance concrete specimens were characterized with 3D Focused Ion Beam/Scanning Electron Microscopy (FIB/SEM), and the fluid transport properties was predicted [36]. Zhang et al. [37] studied the law of seepage flow related to PSD using computed tomography (CT) imaging, and the visual pore structures and seepage characteristics were obtained through 3D reconstruction and numerical simulation.

In this study, the concrete materials with strong hydrophobicity are processed combining with low-heat Portland cement and hydrophobic admixture named Yellow River Engineering Consulting (YREC). The hydrophobic modification of concrete through addition of YREC is further analyzed. For the novel hydrophobic agent, the electron laser particle size analysis, scanning electron microscope (SEM) test, chemical composition analysis, and Fourier-transform infrared spectroscopy are used to investigate the properties comprehensively. Concrete specimens of different curing ages were prepared considering three kinds of content. The effect of YREC was evaluated from the four aspects of concrete: defect degree, strength, impermeability, and hydration products. The multi-scale pore structure changes of mortar matrix induced by YREC were further discussed using low-temperature liquid nitrogen adsorption (LT-NA) and MIP.

## 2. Materials and Methods

### 2.1. Hydrophobic Admixture Preparation

The hydrophobic admixture named YREC was selected to prepare the concrete specimens. YREC admixture is a kind of hydrophobic powder with flake inorganic material as the base material, which is processed by crushing, particle control and chemical modification. Mica powder as the substrate of YREC, comprised mainly of SiO_2_ and Al_2_O_3_, was dried in an oven at a temperature of 105 °C for 48 h. Next, the dried mica was ground in a mill and sieved using a negative pressure sieving method with a 2.5 μm thickness. Finally, a mixture composed of 98% mica, 1% silane coupling agent (KH550), and 1% polydimethylsiloxane (PDMS) by mass were added to a powder surface modification machine and processed at a temperature of 60 °C for 20 min. The specific preparation process was also introduced in our previous work [38,39]. Laser particle size analysis was performed on the YREC powder directly. The microstructure of YREC admixture was analyzed by SEM with 15 Kv acceleration voltage. The chemical composition of YREC was analyzed by LABCENTER XRF-1800 fluorescence spectrometer using dried YREC powder directly. Fourier-Transform Infrared Spectroscopy (FTIR) was also run on the YREC to evaluate the functional groups applying pressed-disk technique. The 1–2 mg YREC powder was finely mixed with 200–400 mg dried potassium bromide, and ground the powder until the particle size was about 200 mesh. We took out about 100 mg mixture and put it in a clean pressing mold, then press it for 1–2 min under 20 MPa in the tablet press. Until pressed into a transparent sheet, it could be used for determination.

### 2.2. Concrete Specimens

The cement material, P•LH 42.5 was obtained from the Huarun Company (Henan, China). Fly ash with the specific gravity of 2360 kg/m^3^ was adopted. Fine aggregate was obtained from machine sand, which had a fineness modulus of 2.78. The coarse aggregate was crushed basalt (4–20 mm), and the apparent density was 2780 kg/m^3^. The size of specimens is 100mm × 100mm × 100mm. The value of water-cement ratio is 0.50. The specimen without YREC was named L, and the specimens mixed with YREC were named LY, LY-2, LY-4, and LY-6 represented the 2%, 4%, and 6% YREC addition, respectively. Five different cueing ages were designed (3, 7, 14, 28 and 90 days) with 95% relative humidity curing environment and a temperature of 20 °C. The mix proportions of all the concrete were listed in Table 1. The mechanical properties were tested using TAW-2000 rock testing machine (Jinli Test Technology Co., Ltd., Jilin, China).

### 2.3. Hydrophobic Tests

For permeation test, the specimens at 7, 14, 28 and 90 days were carried on the HP.4.0 full-automatic concrete anti-permeability apparatus. The water pressure started from 0.1 MPa and increased by 0.1 MPa every 8 h [40]. For contact angle test, the specimens at 28 days were taken out of the standard curing room, and the contact angle between the concrete surface and water was tested after the surface was naturally air-dried.

### 2.4. Ultrasonic Tests

In a complete concrete structure, the sound wave has high amplitude. It will decay rapidly and directly affect directly the propagation speed of the wave when it encounters pores or cracks. This feature can be used to evaluate the defects of concrete. In addition, ultrasonic non-destructive test was carried out on 100 mm cubic specimens to evaluate the defect degree of concrete. The relative defect degree *D_c_* was proposed to characterize the defects inside the concrete, which was expressed as:

(1) $$D_{c}=1-{\left(V_{t}/V_{90}\right)}^{2}$$

where *D_c_* was the relative defect degree, *V_t_* was the wave velocity of concrete at *t* days (km/s), which satisfied *t* < 90, *V*_90_ was the wave velocity of concrete at 90 days (km/s).

### 2.5. Multi-Scale Pore Tests

LT-NA was carried on the ASAP 2020 physical adsorption device (Micromeritics Instrument Corp., Norcross, GA, USA) at Tinghua University. Before LT-NA measurements, all particles were dried for 24 h at a constant temperature of 60 °C. The PSD of mortar matrix was obtained using the Barrett–Joyner–Halenda (BJH) model according to N_2_ adsorption/desorption. The mercury intrusion measurements were conducted using an AutoPore IV9500 Micrometrics Instrument (Micromeritics Instrument Corp., Norcross, GA, USA). The mortar matrix specimens from L and LY groups were selected, and each particle size is between 5 and 10 mm at 28 days.

The advantages of fractal theory in surface analysis were first introduced in 1983 [41,42]. The Frenkel–Halsey–Hill (FHH) model is considered to be effective and reliable for a wide range of porous materials [43,44]. In this study, the FHH model was used to evaluate the roughness and complexity of pore surface and expressed as:

(2) $$\ln(\frac{V}{V_{0}})=\alpha \ln\left(\ln\left(\frac{P_{0}}{P}\right)\right)+\delta$$

where *V* is the volume of adsorbed gas molecules at the equilibrium pressure *P*, *V*_0_ is Monolayer adsorption volume, *P*_0_ is the gas saturation pressure, *α* is the coefficient associated with a fractal value, *δ* is the y-intercept in the log-log plot. The log-log plots of ln(*V/V*_0_) vs. ln(ln(*P*_0_/*P*)) can be obtained from the LT-NA data [44,45,46]. The surface fractal dimension is calculated as:

(3) D=α+3

and

(4) D=3α+3

### 2.6. Hydration Product Test

X-ray powder diffraction (XRD) (Bruker D8 Advance, Bruker German, Leipzig, Germany) was used to test hydration product. The powder of cement paste from L, LY-2, LY-4, and LY-6 at 28 days was selected. The scanning range was 5–70°, and step size was 0.02°.

## 3. Results and Discussion

### 3.1. Microscopic Characteristics and Elemental Composition Analyses of YREC

**Figure 1:**
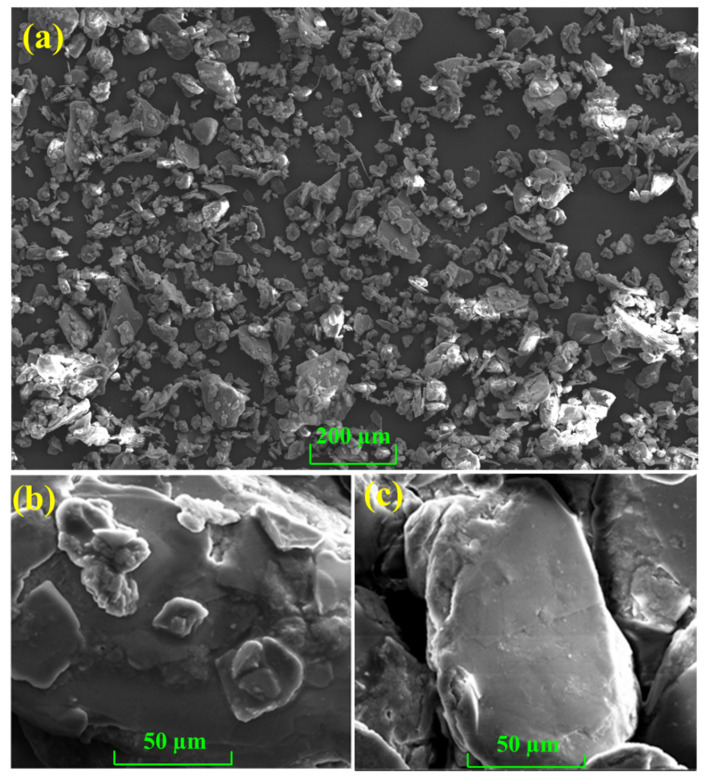
SEM results for the Yellow River Engineering Consulting (YREC): (**a**) the overall morphology; (**b**) multilayer platform structure; (**c**) flat structure.

The YREC particles mainly distributed around 90 μm. The particles less than 25 μm accounted for 10%, and the particles larger than 195 μm accounted for 10%. The dominant particles ranging from 25 to 195 μm accounted for 80%. The need for properly characterizing the microstructure of YREC will help in explaining the materials work and the interaction with concrete. The microstructure of YREC admixture using SEM is shown in Figure 1. Two morphologies were observed in YREC at high magnification. The dominant structure of YREC was the multilayer platform structure (Figure 1b), accounting for about 80%. The size was widely distributed ranging from 2–300 μm. The typical characteristic of multilayer platform structure was layer by layer. The content of flat structure was about 15% with a size of 50–200 μm, and the surface was relatively smooth (Figure 1c). The hydrophobicity induced by YREC relies on the analysis of the characteristics of their constituents. The chemical compositions of YREC are listed in Table 2.

**Figure 2:**
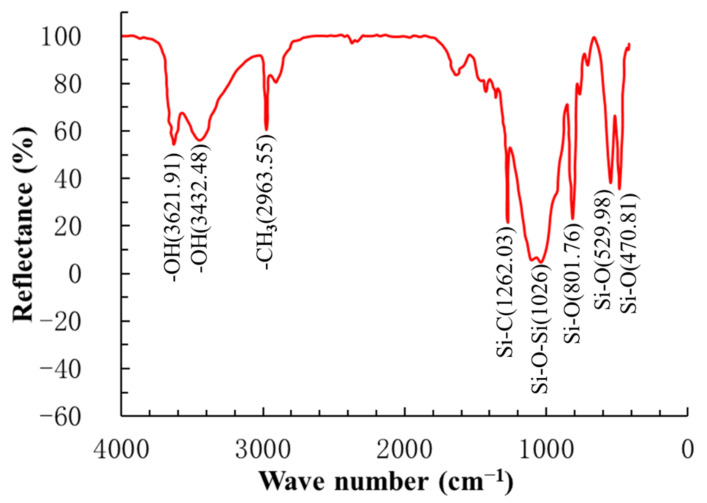
Fourier-Transform Infrared Spectroscopy (FTIR) analysis for the YREC.

FTIR spectrum was chosen to run an analysis on the functional groups of YREC. This technique is well known for its high sensitivity, accuracy, and reliability in analyzing materials [45]. The FTIR spectrum of YREC is shown in Figure 2. The typical functional groups were recognized with some characteristic peaks at 3621.91, 3432.48, 2963.55, 1262.03, 1026, 801.76, 529.98, and 470.81 cm^−1^. The functional groups corresponding to each wave peak are also shown in the Figure 2. The most of functional groups obtained from FTIR were hydrophobic, which mixed in cement-based materials could provide a certain amount of water resistance.

### 3.2. The Improvement of Impermeability Induced by YREC

**Figure 3:**
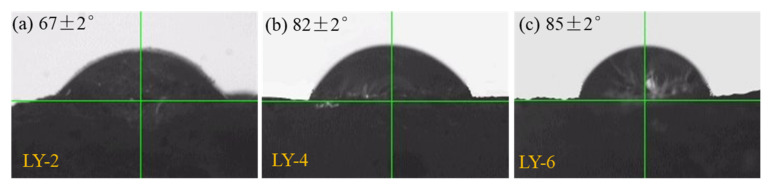
Contact angles of concrete at 28 days: (**a**) LY-2; (**b**) LY-4; (**c**) LY-6.

**Figure 4:**
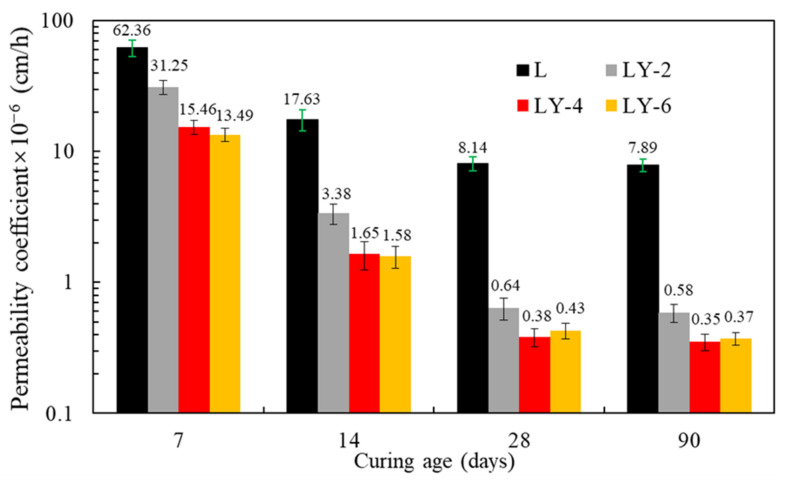
Water permeability of concrete with different curing ages and YREC content.

Figure 3 is a typical contact angle test diagram of the YREC concrete surface contacted with water. The contact angles of LY-2, LY-4, and LY-6 were 67 ± 2°, 82 ± 2° and 85 ± 2°, respectively. The contact angle increased with the increase of YREC addition. When the content increased to 4%, the content of organosilicon continued to increase, and the contact angle could not continue to increase significantly. When the content increased to 4%, the contact angle could not be significantly increased by mixing more YREC. The water permeation results are shown in Figure 4, which could effectively evaluate the water resistance of concrete. The higher value of permeability coefficient represents the poorer hydrophobicity. The impermeability of LY-2 was doubled at 7 days compared with L. Moreover, with the increase of curing days, the hydrophobic effect of YREC got better, which was up to more than 20 times higher than that of ordinary concrete. The impermeability of LY-4 and LY-6 were similar at various curing ages. When the admixture was 4%, the role of YREC could be fully played.

### 3.3. Mechanical Properties and Internal Defect

**Figure 5:**
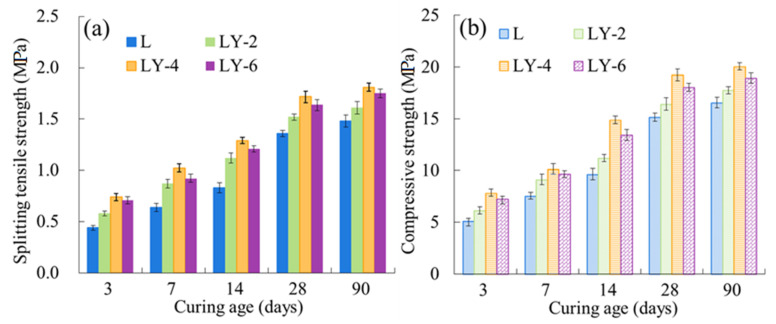
Mechanical properties of L and LY: (**a**) spitting tensile strength and (**b**) compressive strength.

Splitting tensile strength and compressive strength of concrete specimens in L and LY groups with 3, 7, 14, 28 and 90 curing days were shown in Figure 5. With the increase of curing days, both the splitting tensile strength and compressive strength improved, and the strength of the LY specimen was always higher than that of L specimen. It’s obvious that the addition of YREC improves the strength of concrete significantly. For instance, when the curing age reached 28 days, the maximum splitting tensile strength of LY specimen was 1.72 MPa (LY-4), while the splitting tensile strength of L specimen was only 1.36 MPa. YREC improved the splitting tensile strength of concrete by nearly 30%. Moreover, the strength order of specimens with YREC was LY-4 > LY-6 > LY-2. Both the splitting tensile strength and compressive strength were the highest when the YREC content was 4%, and with 90 curing days, the splitting tensile strength of LY-6 and LY-2 were 96.69% and 88.95% of LY-4, respectively.

**Figure 6:**
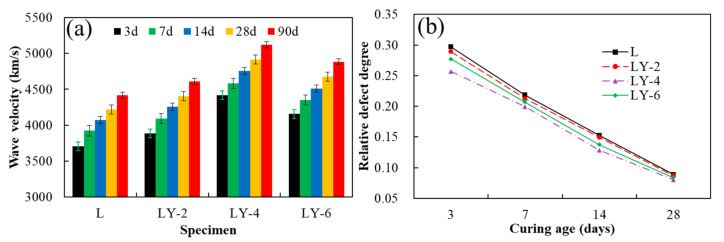
Ultrasonic non-destructive test results: (**a**) wave velocity, (**b**) relative defect degree.

Figure 6 shows the ultrasonic non-destructive test results including wave velocity and relative defect degree. In Figure 6a, the wave velocities of L specimen under any curing ages are lower than that of LY specimen. At 28 days, the wave velocity of LY-4 was 4913 km/s, while that of L was only 4218 km/s. Moreover, the wave velocity of LY-6 was lower than that of LY-4 and higher than that of LY-2 at various curing days. The results corresponded to the mechanical properties well, which could be understood that the ultrasonic non-destructive test results could reflect the interior defects of concrete such as pores and micro cracks. In Figure 6b, the relative defect degree *D* decreased with curing days, which represents the improvement of the internal density of the concrete correspondingly. The relative defect degree of L was higher than that of LY, and the difference became smaller when the curing age reached 28 days, but LY specimens were still higher, which indicated that YREC improved the internal density obviously, especially at early times. The relative defect degree *D* of LY-4 was the lowest in the LY specimen.

**Figure 7:**
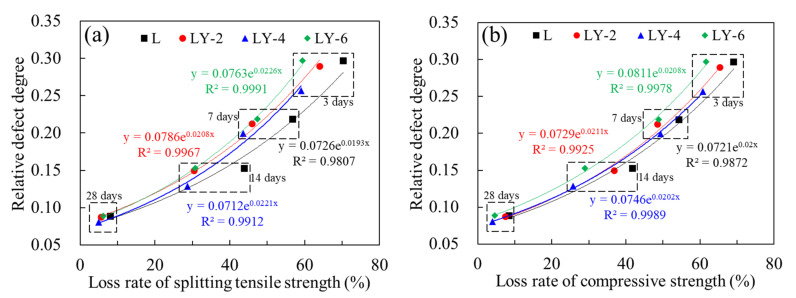
Relationship between relative damage degree and loss rate of strength: (**a**) splitting tensile strength and (**b**) compressive strength.

Since the relative defect degree *D_c_* was closely related to the mechanical properties, the relationship considered the influence of YREC content and curing ages is further analyzed. The results are shown in Figure 7, where loss rate of splitting tensile strength is defined as Equation (5). The loss rate of compressive strength defined in the same way is expressed as Equation (6).

(5) $$L_{ts}=\frac{f_{ts-90}-f_{ts-t}}{f_{ts-90}}\times 100$$

where *L*_*ts*_ is the loss rate of splitting tensile strength (%), *f*_*ts-t*_ is the splitting tensile strength under *t* curing days (km/s), which satisfied *t* < 90, *f*_*ts*−90_ is the splitting tensile strength with 90 curing days (MPa).

(6) $$L_{c}=\frac{f_{c-90}-f_{c-t}}{f_{c-90}}\times 100$$

where *L*_*c*_ is the loss rate of compressive strength (%), *f*_*c−t*_ is the compressive strength under *t* curing days (km/s), which satisfied *t* < 90, *f*_*c*−90_ is the compressive strength with 90 curing days (MPa).

It could be seen that the relative defect degree *D_c_* met the exponential relationship with the loss rate of both splitting tensile strength and compressive strength, and the correlation coefficient *R*^2^ arranges from 0.09807 to 0.9991. It indicates that the characterization method of relative defect degree proposed in this paper is available to evaluate the mechanical properties of concrete. The loss rate of both splitting tensile strength and compressive strength could be obtained through these ultrasonic non-destructive test results, thus the long-term strength of concrete can be predicted based on the early strength, which was of great significance in engineering scale.

### 3.4. Pore Structure Characteristics

#### 3.4.1. Results From MIP

**Figure 8:**
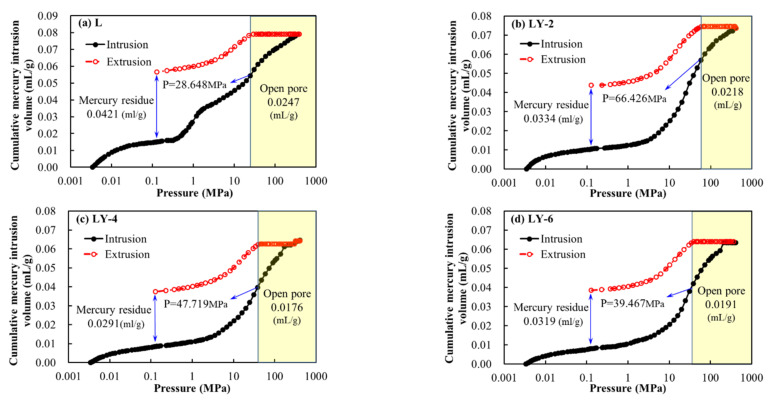
Mercury intrusion and extrusion curves of samples: (**a**) L; (**b**) LY-2; (**c**) LY-4; (**d**) LY-6.

The MIP analyses are widely accepted to be capable to characterize a large scale of pore size, especially for macro-pores in the porous media. The porosities of L, LY-2, LY-4, and LY-6 were 18.20%, 16.08%, 14.13%, and 14.51%, respectively. The whole intrusion/extrusion processes of MIP show that there were different degrees of hysteresis loops in specimens, among which the hysteresis of L specimen was more serious than that of LY-2, LY-4 and LY-6 (Figure 8). It is generally believed that the hysteresis loop is dominated by the shape, clustering, and connectivity of the pores, and the characteristics of the hysteresis loop provide certain reference value for the evaluation of pore connectivity. The trend of intrusion cures showed approximate linear growth for L sample YREC (Figure 8a). The intrusion cures show the “S” shape for the samples mixed with YREC (Figure 8b,c). It indicates that the samples without YREC had the better pore connectivity, especially for small pores corresponding to the intrusion pressure larger than 10 MPa. For extrusion curves, all of the samples had the horizontal phase. There was no mercury comes out of the pores. It was mainly due to lots of open pore volumes. For L sample, the corresponding mercury intrusion pressure at the end of horizontal phase was less than that of the LY samples, which proved that L sample had better pore connectivity again. The quantities of mercury residue also indicated that the mortar matrix samples contained a large number of open pores. In general, the quantity of open pore volumes of L was larger than that of LY-2, LY-4, and LY-6. Both the more quantities of mercury residue (larger open pore volumes) and the better pore connectivity provided favorable conditions for water migration.

#### 3.4.2. N_2_ Adsorption Results and Pore Fractal Characteristics

**Figure 9:**
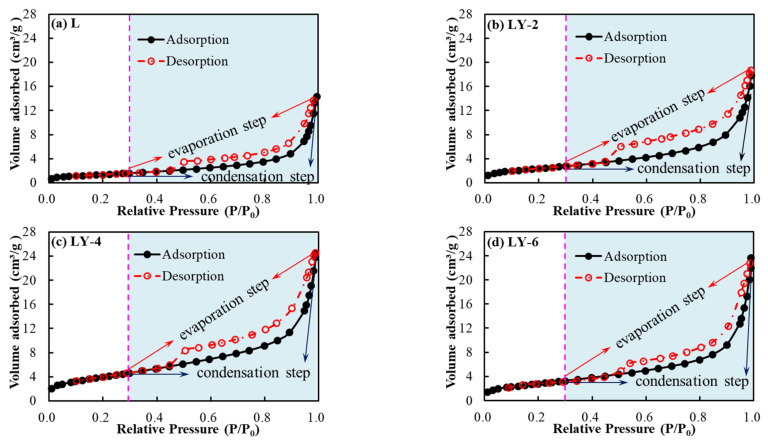
Low-temperature liquid N_2_ adsorption/desorption results from specimens in L and LY groups: (**a**) L; (**b**) LY-2; (**c**) LY-4; (**d**) LY-6.

The isothermal adsorption/desorption curves of the four mortar matrix specimens are shown in Figure 9. The saturation adsorption quantity of L sample was the minimum, and it indicated that the small pore volume (pore size < 180) of the sample was small. For LY samples, the values of saturation adsorption quantity were larger than 16 cm^3^/g. The addition of YREC has a filling effect and inhibited the generation of larger pores in the cement hydration process [46,47]. The main reason is that YREC particles have a certain hydrophobicity, which can refine water, and more gel materials can undergo hydration reaction. The content of small pores (pore size < 180 nm) did not increase with the increasing dosage of YREC. The small pore content at 6% was very close to that at 4%. The pore volume of LY specimens obtained by N_2_ adsorption was larger than that of the L sample.

**Figure 10:**
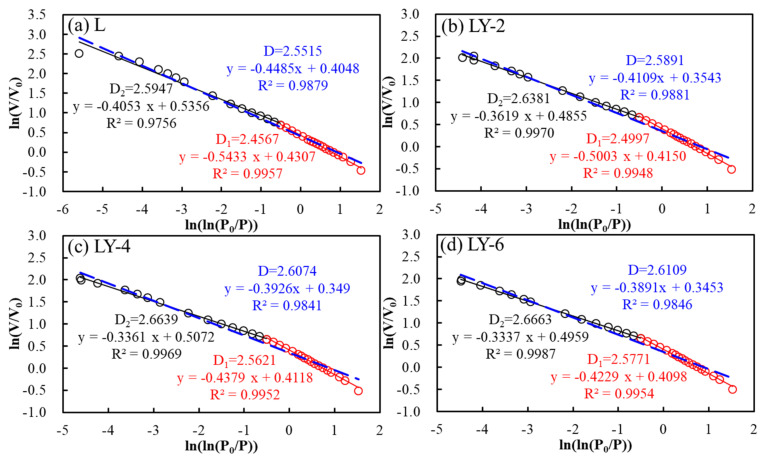
Fractal analysis of N_2_ adsorption isotherms: (**a**) L; (**b**) LY-2; (**c**) LY-4; (**d**) LY-6.

**Figure 11:**
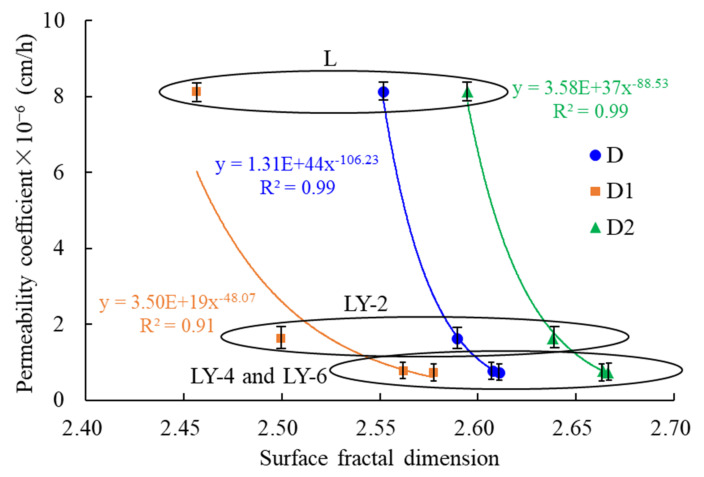
The relationship between fractal dimension and permeability.

As shown in Figure 10, all the points were usually divided into two linear segments at ln(ln(*P_0_*/*P*)) = −0.50. It is necessary to point that the value at −0.50 corresponded to the pores with a diameter of about 5 nm [41]. In order to depict different aspects of pore characteristics, two different linear intervals were expressed as the results of different mechanisms of liquid N_2_ adsorption at low and high pressures respectively. Two fractal dimensions named *D*_1_ and *D*_2_ could be obtained by linear fitting of two parts of data. *D*_1_ corresponded to monolayer coverage in pores with the diameter less 5 nm, and *D*_2_ corresponded to multilayer coverage in the pores > 5 nm. The average fractal dimension (*D*) was obtained from linear regression of the whole pressure segment to evaluate the overall heterogeneity of pore structure. The values of *D*, *D*_1_, and *D*_2_ increased with the increasing addition of YREC, and the L sample with the minimum value indicates that the pore surface structure of LY samples were more complex than that of L sample (Table 3). With the increase of YREC content, the increase amplitude of *D*_1_ was greater than that of *D*_2_, which proved that YREC effectively adjusted the surface roughness of the pore less than 5 nm. In the connected pore network, the micropores determined the permeability, and the more complex micropore structure, the more beneficial to the impermeability. Complex pore surface could improve impermeability of concrete due to longer penetration paths or liquid bypass. In combination with the water permeability, there was a good power function relationship between the surface fractal dimension (*D*, *D*_1_ and *D*_2_) and the water permeability coefficient (Figure 11). The correlation coefficients were all above 0.9, which indicates that fractal analysis could give a preliminary forecast on water permeability.

#### 3.4.3. Multi-Scale PSD Analyses

**Figure 12:**
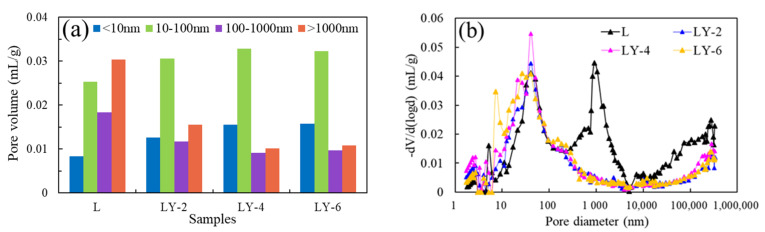
Pore size distribution in combination of mercury intrusion/extrusion porosimetry (MIP) and nitrogen absorption (NA): (**a**) pore volume with different pore size ranges, (**b**) different pore sizes corresponding to incremental pore volume.

Pore volumes obtained from the MIP data and LT-NA results are shown in Figure 12. The pores larger than 100 nm were harmful pores, which provided the channel for water invasion [48,49]. The pores with a diameter larger 1000 nm had an obvious change induced by YREC. For the volumes of mesopores (100–1000 nm), LY samples were much smaller than L samples. YREC had little effect on the transition pore, and the volumes of the transition pore were high in each sample. The volumes of pore size < 10nm in LY samples were 1.5–2 times than that in L sample. The volume of micropores increased with the increase of YREC content. There was no obvious difference between LY-4 and LY-6 for the various pore size ranges (Figure 12a). For the hydrophobicity of cement-based concrete, the advantageous pores were micropores and transition pores, which were not conducive to the migration of water molecules. The harmful pores (macropores and mesopores) of L accounted for 59.05%, which was larger than LY-2 (38.61%), LY-4 (28.54%), and LY-6 (29.82%). As shown in Figure 12b, the comparison of the PSDs indicated that YREC could significantly reduce harmful pores in mortar matrix. For LY samples, when the pore size was larger than 10 µm, the pore volume increased slowly with the pore diameter.

### 3.5. The Effect of YREC on Hydration Reaction

**Figure 13:**
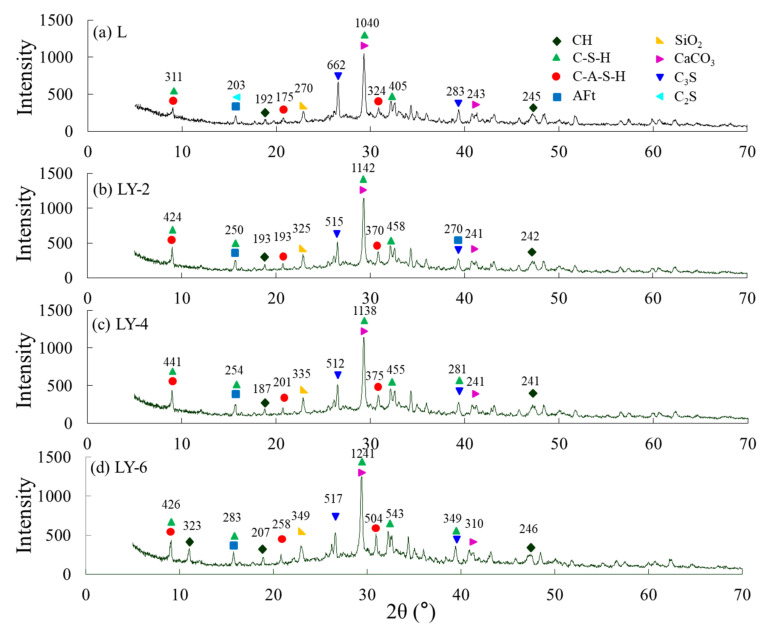
XRD patterns of samples at 28 days: (**a**) L; (**b**) LY-2; (**c**) LY-4; (**d**) LY-6.

XRD patterns of L, LY-2, LY-4, and LY-6 at 28 days are shown in Figure 13. The main formation of CaCO_3_ and C–S–H was detected at 28–29° with the highest diffraction intensity. For L sample, a small amount of C_2_S did not participate in the hydration reaction and was detected at 16.2°. It was different from LY samples, and C–S–H crystal was generated at around 16°. It indicated that the YREC could promote the hydration reaction of the C_2_S with water. With the increase of YREC content, the development of C_3_S crystals was more strongly inhibited due to the peak shape at 26.8° for four samples. For LY-4 and LY-6, the C–S–H was detected at 2θ = 38–39°. Within the same scan (range2θ = 38–39°), C_3_S and Aft were well-developed in L and LY-2. The results once again showed that YREC promoted hydration. Moreover, the calcium hydroxide crystal (CH) of LY-4 developed better at 11.3° according to Figure 13a. Wang et al. [50] and Haruehansapong et al. [51] reported that the development of CH crystal led a negative effect on the strength. Han et al. [52] discovered that the refinement of CH crystal size and quantity could further improve the interface transition zone, and thus enhance the concrete compactness and strength. It is the reason that the strength of LY-4 was higher than that of LY-6. Excessive content of YREC could lead to the development of CH crystal.

## 4. Conclusions

A method to improve the workability of concrete using YREC was explored. With this approach, the hydrophobicity, strength and compactness of the derived concrete were promoted. Several results are reached:

1. The components of YREC and a large number of hydrophobic functional groups provided strong hydrophobicity. The structure of YREC particles is favorable for impermeability due to the complex and longer seepage path in concrete based on fractal analysis and hydrophobic tests.
2. The defect degree of concrete can be deduced by YREC, and the splitting tensile strength and compressive strength were improved significantly due to the filling effect. The addition of 4% YREC is the most desirable method, which can promote the hydration reaction and further enhance the functionality of concrete.
3. The porosity of the mortar matrix materials decreased with the addition of YREC, which inherently affected the diffusion of water. YREC can restrain the formation of harmful pores in concrete mortar matrix, which mainly decreased the pore volume with the pore size larger than 10 µm. Moreover, the more advantage pores (micropores and mesopores) developed in concrete mortar matrix with the addition of YREC.
4. YREC can significantly improve the hydrophobicity of concrete materials. The next step is to study the properties of YREC in acidic environment or chlorine salt and sulfate environments. The application range of the material is desirable to further investigated.

## Figures and Tables

**Table 1:** Mix proportions of concrete mixes (kg/m^3^).

Mix Designation	Water	Cement	Fly Ash	Sand	Gravel	YREC	Water-Binder Ratio
L	130.00	169.00	91.00	727.58	1351.23	0	0.5
LY-2	130.00	169.00	91.00	727.58	1351.23	6.50	0.5
LY-4	130.00	169.00	91.00	727.58	1351.23	13.00	0.5
LY-6	130.00	169.00	91.00	727.58	1351.23	19.50	0.5

**Table 2:** Chemical composition from fluorescence spectrum analysis.

Chemical Component	SiO_2_	Al_2_O_3_	K_2_O	Fe_2_O_3_	MgO	Na_2_O	SO_3_	CaO	Others
Content (%)	62.3696	20.4022	7.5609	5.5509	1.0453	0.8712	0.6152	0.2689	0.6764

**Table 3:** Fractal dimension values for L and LY group samples.

	L	LY-2	LY-4	LY-6
*D*	2.5515	2.5891	2.6074	2.6109
*D_1_*	2.4567	2.4997	2.5621	2.5771
*D_2_*	2.5947	2.6381	2.6639	2.6663

## Data Availability

Data is contained within the article.
